# P-330. Beneath the PPE: Perspectives on Infection Prevention from Emergency Department Healthcare Personnel

**DOI:** 10.1093/ofid/ofae631.533

**Published:** 2025-01-29

**Authors:** Laya Dasari, Julio Ma Shum, Eileen F Searle, Molly Paras, Erica S Shenoy, Paul Biddinger

**Affiliations:** Massachusetts General Hospital, Boston, Massachusetts; Massachusetts General Hospital, Boston, Massachusetts; Massachusetts General Hospital, Boston, Massachusetts; Massachusetts General Hospital; Mass General Brigham, Boston, MA; Mass General Brigham, Boston, MA

## Abstract

**Background:**

Adhering to best practices in infection prevention and control (IPC) can be challenging in emergency departments (EDs) due to high patient volumes, high acuity of illness, and limited initial information about patients’ history, among other factors. Understanding ED healthcare personnel’s (HCP) perceptions is important for improving IPC practices in this environment.

**Table 1.**
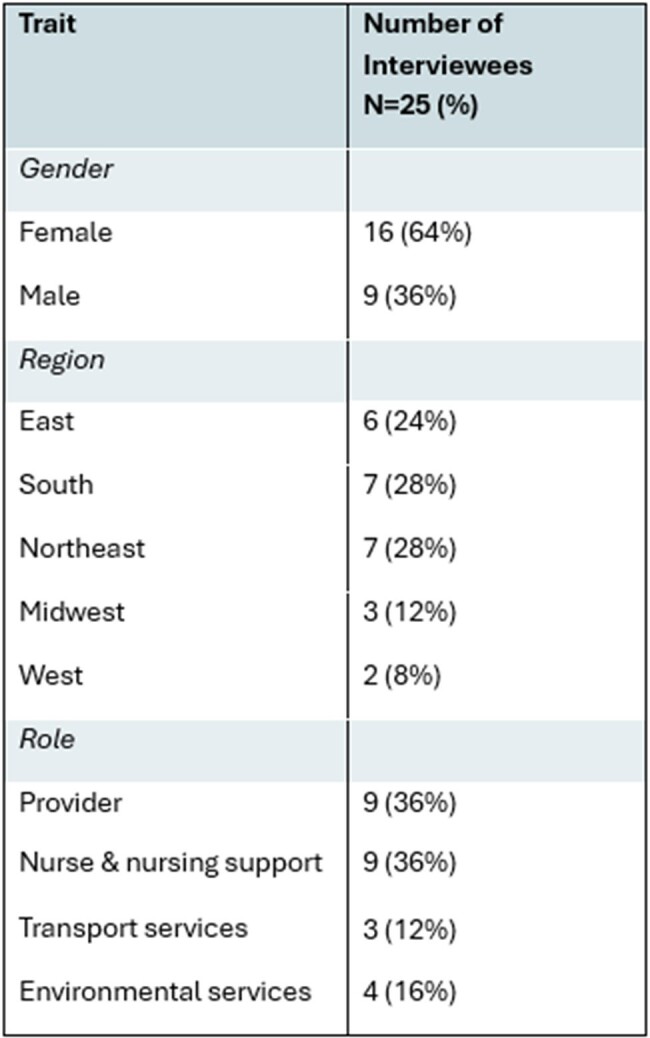
Demographics of interviewees.

**Methods:**

Between August 2023 and November 2023, we conducted semi-structured interviews focused on IPC with HCP working in emergency departments across the United States. EDs were selected for inclusion via convenience sampling from a larger group of EDs selected for representative geographic, volume, and practice type variety. Our study solicited voluntary participation from staff in these EDs via their ED leaders. Each interview was recorded, transcribed, and subsequently coded across 19 themes by two independent raters. Each theme as sorted as either a facilitator or barrier to following recommended IPC practices in the ED. We then used content analysis to quantify the frequency of the identified themes, and stratified responses by role group.

**Table 2.**
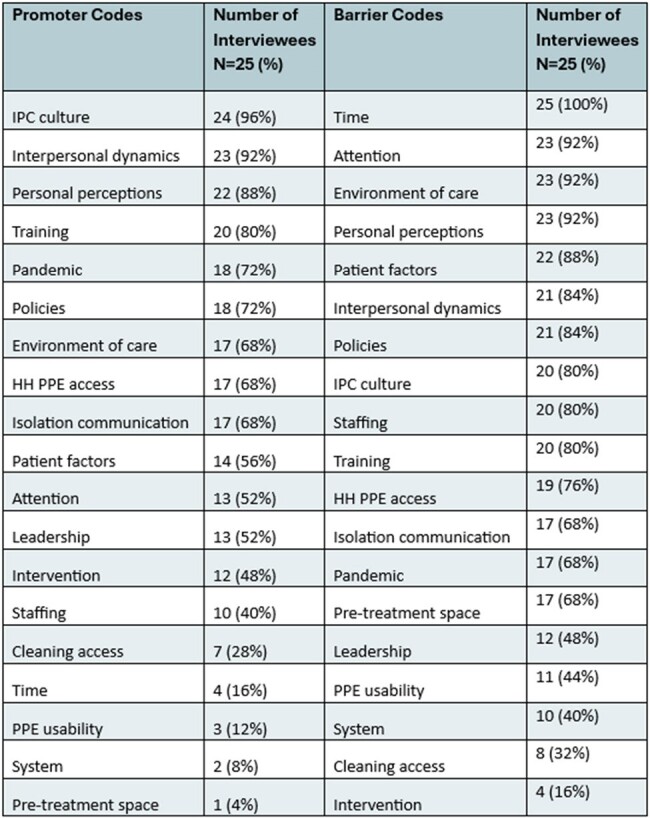
All identified codes and frequencies in interviews.

Codes identified in all interviews, sorted into promoters and barriers. Higher percentage is indicative of more mentions of each code across all interviews. Note: IPC = infection prevention and control; HH = hand hygiene; PPE = personal protective equipment.

**Results:**

We conducted 25 interviews across four role groups (Table 1). Key barriers identified in the interviews included constraints related to time (25/25, 100%), attention (23/25, 92%), environment of care (23/25, 92%), and personal perceptions (23/25, 92%), while top promoters included IPC culture (24/25, 96%) and interpersonal dynamics (23/25, 92%) (Table 2). Stratified analyses indicated that there were variations among roles, with environmental staff emphasizing the importance of training, and nurses highlighting policies both as a promoter and barrier (Table 3).

**Table 3.**
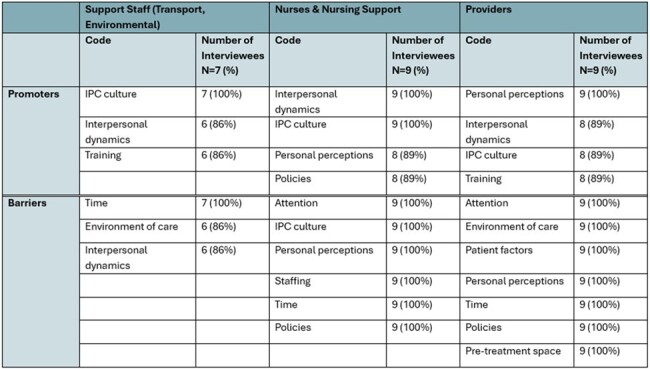
Top codes stratified by role.

Higher percentage is indicative of more mentions of each code across each group. Codes are stratified by healthcare personnel’s role (with transport and environmental services staff pooled to support staff) and promoter/barrier categorization. Note: IPC = infection prevention and control.

**Conclusion:**

The main barriers to effective IPC measures perceived by ED staff are limited time and other demands on their attention in the ED environment. A strong culture promoting IPC and cohesive team dynamics were the primary IPC facilitators, suggesting potential targets for future interventions. Future research should explore interpersonal relationships further by conducting comparative studies across different types of hospital emergency departments, and longitudinally while testing strategies to improve IPC adherence.

**Disclosures:**

**All Authors**: No reported disclosures

